# Association between Growth Hormone-Insulin-Like Growth Factor-1 Axis Gene Polymorphisms and Short Stature in Chinese Children

**DOI:** 10.1155/2018/7431050

**Published:** 2018-03-04

**Authors:** Yanhong Zhang, Mei Zhang, Yuntian Chu, Baolan Ji, Qian Shao, Bo Ban

**Affiliations:** ^1^Qingdao University, Qingdao 266200, China; ^2^Department of Endocrinology, Affiliated Hospital of Jining Medical University, Jining, Shandong 272029, China

## Abstract

**Objective:**

This study was designed to analyze the association between the growth hormone-insulin-like growth factor-1 (GH-IGF-1) axis gene polymorphisms and short stature in Chinese children.

**Methods:**

181 growth hormone deficiency (GHD) patients and 206 normal stature controls were enrolled to attend this study. Five single-nucleotide polymorphisms in the GH receptor (GHR) and 5 SNPs within the GH-signaling pathway were genotyped by matrix-assisted laser desorption/ionization time of flight mass spectrometry. We conducted an association study between these SNPs and the risk of developing short stature. Linkage disequilibrium analysis was performed using Haploview software and the associations of the SNPs frequencies with short stature were analyzed using* X*^2^ tests.

**Results:**

No significant difference was found in gender, weight, height, and BMI between the GHD and control groups, except that the age of GHD group was older than the control one. Allele and genotype frequencies were consistent with those expected from Hardy-Weinberg equilibrium. Compared with the controls, heterozygous genotype frequencies (CT) of rs12515480 and rs6873545 of GHR gene were significantly lower. Genotype frequencies of the other 8 SNPs did not show significant difference between these two groups. Considering a dominant model, an OR < 1 was observed for genotypes rs12515480 (OR = 0.532, *P* = 0.019) and rs6873545 (OR = 0.587, *P* = 0.017).

**Conclusions:**

The heterozygous genotypes of rs12515480 and rs6873545 of GHR gene were associated with decreased risk of GHD in Chinese children.

## 1. Introduction

Short stature is a common medical concern in childhood which is characterized by a height more than 2 standard deviations (SD) below the mean for a given age, gender, and population group. Human height depends on complex factors including parents' heredity, genetic mutation, intrauterine infection, nutrition status, hormonal influences, and social environment [1]. Although researchers have identified several causes of short stature which are including familial short stature, hormonal pathologies, systemic diseases, birthday history of intrauterine growth retardation (IUGR), or small for gestational age (SGA) [2]. However, the underlying causes remain unknown in approximately 80% of patients and are therefore classified as idiopathic short stature (ISS) [3].

Growth hormone deficiency (GHD) is a relatively common disorder with a prevalence of approximately 1 in 4000 during childhood, which is one of the major causes of short stature [4]. Our previous studies showed that the five most common etiological factors of short stature were as follows: GHD (55.56%), ISS (30%), hypothyroidism (4.07%), SGA (2.59%), and congenital ovarian hypoplasia (2.59%) [5]. The five most common etiological factors from the study by Sultan et al. [6] were constitutional growth delay (CGD), familial short stature (FSS), malnutrition, coeliac disease, and GHD. Rabbani et al. [7] carried the similar study and also found that GHD was one of the most common causes of short stature. The above information suggests that it would be critical to explore the pathogenesis of GHD.

Short stature is considered as a genetic origin disease, as shown by identification of genes deficiency associated with short stature, such as short stature homeobox (SHOX), insulin-like growth factor-1 (IGF-1), signal transducer and activator of transcription 5B (STAT5B), insulin-like growth factor binding protein acid subunit (IGFALS), and insulin-like growth factor-1 receptor (IGF1-R). The genome-wide association studies (GWAS) using single-nucleotide polymorphisms (SNPs) analysis have revealed a large number of genetic variants associated with the height, and 75% of the height could be explained by the inheritance [8], and 180 human genetic loci were found to be related to height, but it can only explain about 10% of the height variation in the GIANT alliance research [9]. Thus, it suggested that we need to put more efforts into identifying novel genes variants associated with short height.

Traditionally, the growth hormone-insulin-like growth factor-1 (GH-IGF-I) axis is the most important signaling pathway in linear growth, and defects in this axis present as growth hormone deficiencies or IGF-I deficiencies [10]. There were various genes and SNPs associated with short stature, including GHR SNPs (rs10044169, rs12515480, rs4410646, rs6182, and rs6873545), IGF-1R SNPs (rs1976667, rs2684788), IGFALS SNPs (rs17559, rs3751893), and IGFBP-3 SNP (rs2132570) [11]. However, the above gene polymorphisms have not been elucidated systematically among Chinese GHD children. Therefore, the aims of this study were to explore the GHD mechanism from the molecular genetic perspective of the GH-IGF-1 axis, to screen SNP locus related to GHD genetic susceptibility, and to analyze the relationships between SNPs of the GH-IGF-1 axis gene and GHD genetic susceptibility.

## 2. Subjects and Methods

### 2.1. Subjects and Grouping

Short stature patients were voluntary enrolled from the Department of Endocrinology, Affiliated Hospital of Jining Medical University, between May 2013 and April 2016. Normal height control group was recruited from the pediatric clinic during the same period. The subjects were all from the same race. The short stature patients were all evaluated for GH serum levels after two provocative tests (with arginine or L-dopa or insulin). Inclusion criteria for GHD were as follows: (1) the height was lower than the same age, sex, and the 2 standard deviations (SDs) below the population average height of the same race; (2) peak GH levels < 10 ng/ml in both of the two GH stimulation tests; (3) the blood routine examination, liver function, kidney function, thyroid function, and the blood fat were normal; (4) without significant serious psychological and emotional disorders; (5) with normal chromosome and pituitary magnetic resonance; (6) without immunocompetent diseases (i.e., human immunodeficiency virus infections). Familial short stature and physical developmental delay of adolescence were excluded for all subjects. Finally, 181 GHD patients and 206 normal height controls were included in our study. The study was approved by the Human Ethics Committee of the Affiliated Hospital of Jining Medical University (Shandong, China). All of the families of the patients were informed of the aims of the study and the parents gave informed consents.

### 2.2. Anthropomorphic Measurements

The height was measured by a specially designated individual using the same measuring instrument (produced by Nantong Best Industrial Co. Ltd., Jiangsu, China) in the morning, and the allowable error range is 0.1 cm. Height was expressed as the height standard deviation score (SDS) based on normative values for Chinese children [12]. BMI was calculated as the ratio between body weight in kilograms and height in meters squared. The stage of puberty was assessed by physical examinations according to Tanner and Whitehouse [13]. The following criteria were considered as prepuberty [14, 15]: girls with no breast development and no pubic hair and boys with a testicular volume of less than 4 ml and no pubic hair. The bone age (BA) was measured by taking X-ray of the left hand, including the hand bone, wrist, and radial ulnar stem 3-4 cm. The same specially assigned investigator scanned the image and evaluated the BA according to the Greulich-Pyle method [16].

### 2.3. Laboratory Measurements

To assess GH secretion, L-dopa (Levodopa Tablets®, He Feng, Guang Xi, China, body weight more than 30 kg, 500 mg of levodopa; less than 30 kg, 250 mg of levodopa) and insulin (Insulin Injection®, Wan Bang, Jiang Su, China, 0.1 U/kg) were administered orally or subcutaneously injected after overnight fasting. Blood samples were collected 0, 30, 60, 90, and 120 min later to obtain the serum GH concentration for each time point. GH was measured using an chemiluminescence method (ACCESS2, Beckman Coulter, USA) with an analytical sensitivity of 0.010 ug/L. Serum IGF-1 and IGFBP-3 levels were measured by chemiluminescence immunometric method (DPC IMMULITE 1000 analyzer, SIEMENS, Germany) with intra- and interassay CV for IGF-1 of 3.0% and 6.2%, respectively, and intra- and interassay CV for IGFBP-3 of 4.4% and 6.6%, respectively. The liver functions, including alanine aminotransferase (ALT), AST and gamma-glutamyl transferase (GGT), kidney function, including Cr, blood urea nitrogen (BUN), and UA, lipid profiles, TC, high density lipoprotein-cholesterol (HDL-C), LDL-C, triglycerides (TG), and fasting plasma glucose (FPG) were tested by a biochemical autoanalyzer (cobas c 702, Roche; Shanghai, China); thyroid function, including free T3 (FT3), free T4 (FT4) and thyroid stimulating hormone (TSH), gonadotropin, cortisol rhythm, and adrenocorticotropic hormone (ACTH) were tested by a luminescence immunoassay system (cobas e 602, Roche; Shanghai, China).

### 2.4. SNP Selection and Genotyping

Genomic DNA was isolated from whole blood using the DNeasy Blood and Tissue Kit (Qiagen). The following genes and SNPs were selected for analysis: GHR SNPs (rs10044169, rs12515480, rs4410646, rs6182, and rs6873545), IGF-1R SNPs (rs1976667 and rs2684788), IGFBP-3 SNP (rs2132570), and IGFALS SNPs (rs17559 and rs3751893). After the polymerase chain reaction (PCR) and purifying of the product, we used matrix-assisted laser desorption ionization time of flight mass spectrometry (MALDI-TOF MS) methods to detect the sample.

### 2.5. Statistical Analysis

All of the statistical analyses were performed with the R statistical software (https://www.r-project.org) and EmpowerStats (https://www.r-project.org, X&Y solutions, Inc. Boston MA). Normally distributed variables were expressed as mean ± standard deviation (SD); qualitative data were expressed as numbers and percentage (*n*, %). An independent samples *t*-test was used to compare continuous variables between the two groups. The chi-squared test was used to analyze differences between the control and GHD groups in SNP genotypes of the GH-signaling related genes, alleles, and dominant and recessive modes. Linkage disequilibrium calculation and haplotype frequencies determination were performed with the Haploview software (Center for Human Genetic Research, Massachusetts General Hospital, and the Broad Institute of Harvard and MIT). *P* values (2-tailed) less than 0.05 were considered statistically significant.

## 3. Results

### 3.1. Comparison of Clinical Characteristics of Short Stature Patients and Normal Control Group

In this study, we enrolled total 387 subjects, including 181 GHD patients (GHD group) and 206 subjects with normal height (control group). The clinical characteristics of the subjects are shown in Table 1. The mean age in control group was younger than that in the GHD group (*P* < 0.001). The height of controls was slightly higher than that in GHD group (*P* = 0.287). In addition, the BMI in GHD group was slightly lower than that in control group (*P* = 0.980).

### 3.2. The Genotype Distribution Frequency of Ten SNPs Loci of GH-IGF-1 Related Genes in the Study Population

Table 2 summarizes the genotype distribution frequency of ten SNPs loci of GH-IGF-1 related genes in the study population. The Hardy-Weinberg equilibrium test showed no significant difference in ten SNP loci between the GHD group and the normal population (*P* > 0.05). Furthermore, we compared the differences in allele frequencies distribution of ten reported SNPs including GHR SNPs (rs10044169, rs12515480, rs4410646, rs6182, and rs6873545), IGF-1R SNPs (rs1976667 and rs2684788), IGFBP-3 SNP rs2132570, and IGFALS SNPs (rs17559 and rs3751893) in GHD and control group. The results showed that compared with the control group, heterozygous genotypes of rs12515480 and rs6873545 were significantly lower in GHD group (*P* = 0.040 and *P* = 0.046, resp.). However, no significant differences in allele frequencies distribution of the other 8 SNPs (rs10044169, rs4410646, rs6182, rs1976667, rs2684788, rs2132570, rs17559, and rs3751893) were observed in GHD and control group.

### 3.3. Association of GHR Polymorphism with Susceptibility to GHD

In order to analyze the association of GH-IGF axis related gene polymorphisms with the risk of short stature, we assessed the association between the rs12515480 and rs6873545 SNPs and the risk of short stature using three genetic models (codominants, dominant and recessive) by unconditional logistic-regression analysis. As shown in Table 3, the crude analysis revealed that the genotype “CT+TT” in rs12515480 from GHR was associated with a decreased risk of GHD under the dominant model (OR = 0.587, 95% CI: 0.364–0.947, *P* = 0.029). Also, we found that the significance still existed after adjusting for gender and age (OR = 0.532, 95% CI: 0.314–0.900, *P* = 0.019). Similarly, the genotype “CC+CT” in rs6873545 from GHR was associated with a decreased risk of GHD under the dominant model (OR = 0.657, 95% CI: 0.445–0.971, *P* = 0.035). And the significance still existed after adjusting for gender and age (OR = 0.587, 95% CI: 0.380–0.908, *P* = 0.017).

## 4. Discussion

In this study, we assessed the association between ten SNPs within GH-signaling and the risk of short stature in China. We found that the “CT+TT” in rs12515480 and “CC+CT” in rs6873545 from GHR was associated with a decreased risk of GHD under the dominant model, presented to be a protective effect on the incidence of GHD.

The GH-IGF-I axis possesses various effects involving height growth, cardiac function, and behavioral psychology [17]. Many factors could affect the role of this axis [1], and the heredity is especially notable [8]. The growth hormone receptor (GHR) exon3 deleted/full-length (d3/fl) polymorphism has been suggested to affect GH sensitivity. Previous studies have confirmed that the GHR d3/fl polymorphism can be studied by TaqMan SNP rs6873545 genotyping [18]. The homozygous mutation of the d3-GHR showed a poor response to GH treatment than carriers of the fl-GHR in GHD adult [19]. However, another research did not show any association between the d3-GHR genotype and decreased sensitivity to GH in ISS children [20]. However, in our study, homozygous mutation of rs6873545 was rare and the heterozygous genotype was relatively higher within GHD group. Furthermore, the correlation analysis showed that “CC+CT” in rs6873545 was negatively related to the GHD. The underlying mechanism is not yet known and needs to be further explored by expanding the sample size or gene function study.

There were few studies about the relationship between the rs12515480 polymorphism and short stature. Zeng et al. [21] found that GHR gene rs12515480 (C/T) polymorphisms were not associated with idiopathic short stature in Yao nationality children of Guangxi province in China. However, in our study, heterozygous genotype of rs12515480 was negatively related to the GHD. The rs12515480 locus is located in the intron region of GHR gene, and we did not find related studies investigating the structure and function of the encoded protein at this locus. Further studies should be taken to explore the relationship and the mechanism.

In our study, the rest 8 SNPs within GH-signal axis were not related to genetic susceptibility of GHD. In consistent with the previous study [22], the result showed that there were no differences in height of SDS, IGF-1, IGFBP-3, and ALS between carriers and nonallele of the common IGFALS gene loci rs17559 and rs3751893 among normal control and ISS children. They suggested that heterozygous IGFALS gene variants could be responsible for short stature in a subset of ISS children with diminished levels of IGF-1, IGFBP-3, and ALS. However, in our study, we did not find any difference in the above gene polymorphisms and genetic susceptibilities to GHD, whereas our results were inconsistent with other studies. Yu et al. [23] found that three SNPs of GHR (rs6182, rs4410646, and rs10044169) are related to genetic susceptibility to ISS. For rs6182 (G/T), genotypes TT and GT showed a decreased risk under T dominant model (OR = 0.624). Genotype AA of rs4410646 presented a lower risk under C dominant model (OR = 0.674). “CC+CA” genotypes of rs10044169 were associated with a decreased risk of ISS under C dominant model (OR = 0.649). Yang et al. [24] revealed that the rs1976667 and rs2684788 loci were significantly associated with genetic susceptibility to ISS. The G allele at the rs2684788 locus was significantly associated with genetic susceptibility to ISS, showing G dominant inheritance. The rs1976667 and rs2684788 loci of the human IGF-1R gene are likely associated with different genetic susceptibilities to ISS in males and females. But the above studies were focused on ISS children; the underlying principle is unclear and needs to be further explored in the subjects with GHD.

There are several limitations to this study. On the one hand, in this correlation study, we could not conclude the causal relationship. On the other hand, other factors that may affect the study have not been adjusted, such as parent height. Furthermore, it is regrettable that some influencing factors including congenital infection (i.e., toxoplasma gondii infection) and nutrition status during fetal and childhood are missing or incomplete. Therefore, subsequent large samples and prospective and comprehensive studies might be needed to verify the results of this study.

In conclusion, this study showed that heterozygous genotypes of rs12515480 and rs6873545 of GHR gene were associated with decreased risk of developing GHD in Chinese children. However, further studies should be conducted to explore and verify the findings and mechanism of the study.

## Figures and Tables

**Table 1:** The clinical characteristics of all study participants.

Characteristic	Control	GHD
*N*	206	181
Gender (*n*, %)		
Female	96 (46.6%)	51 (28.2%)
Male	110 (53.4%)	130 (71.8%)
Age (y)	7.5 ± 3.2	10.2 ± 3.1^*∗*^
Height (cm)	129.9 ± 20.6	127.9 ± 16.1
Weight (Kg)	31.3 ± 15.2	29.5 ± 11.0
BMI (Kg/m^2^)	17.5 ± 3.7	17.5 ± 3.1

Data are mean ± standard deviation. BMI = body mass index. ^*∗*^*P* < 0.05 is considered significant.

**Table 2:** Genotype distributions of GH-IGF axis related gene included in this study.

Gene SNPs	Genotype	Control (*n* = 206)	GHD (*n* = 181)	HWE *P* value
GHR				
rs10044169	AA	161 (78.2%)	148 (81.8%)	0.751
	AC	43 (20.9%)	31 (17.1%)	
	CC	2 (1.0%)	2 (1.1%)	
rs12515480	CC	161 (78.2%)	158 (87.3%)	0.651
	CT	43 (20.9%)	22 (12.2%)^*∗*^	
	TT	2 (1.0%)	1 (0.4%)	
rs4410646	AA	113 (36.0%)	81 (35.4%)	0.330
	AC	152 (48.4%)	120 (52.4%)	
	CC	49 (15.6%)	28 (12.2%)	
rs6182	GG	252 (80.3%)	189 (82.5%)	0.884
	GT	57 (18.2%)	38 (16.6%)	
	TT	4 (1.3%)	2 (0.9%)	
rs6873545	TT	217 (69.1%)	177 (77.3%)	0.635
	CT	91 (29.0%)	46 (20.1%)^*∗*^	
	CC	6 (1.9%)	6 (2.6%)	
IGF1R				
rs1976667	AA	115 (55.8%)	83 (45.9%)	0.957
	AG	77 (37.4%)	83 (45.9%)	
	GG	14 (6.8%)	15 (8.3%)	
rs2684788	CC	55 (26.7%)	49 (27.1%)	0.886
	CT	96 (46.6%)	88 (48.6%)	
	TT	55 (26.7%)	44 (24.3%)	
IGFBP3				
rs2132570	GG	134 (65.0%)	121 (66.9%)	0.351
	GT	67 (32.5%)	55 (30.4%)	
	TT	5 (2.4%)	5 (2.8%)	
IGFALS				
rs17559	GG	132 (64.1%)	113 (62.4%)	0.931
	GA	68 (33.0%)	61 (33.7%)	
	AA	6 (2.9%)	7 (3.9%)	
rs3751893	GG	116 (56.3%)	103 (56.9%)	0.195
	GA	77 (37.4%)	58 (32.0%)	
	AA	13 (6.3%)	20 (11.0%)	

GHD, growth hormone deficiency; SNP, single-nucleotide polymorphism; ^*∗*^*P* < 0.05 is considered significant.

**Table 3:** Genotype effects of rs12515480 and rs6873545 variation.

SNPs	Alleles	OR	95% CI	Best-fitting model	*P* value
rs12515480	*C*/*T*	0.532	(0.314, 0.900)	Dominant	0.019
rs6873545	*T*/*C*	0.587	(0.380, 0.908)	Dominant	0.017

GHD, growth hormone deficiency; SNP, single-nucleotide polymorphism; OR, odds ratio; CI, confidence interval. The model was adjusted for age and sex.
